# A study of using epigenetic modulators to enhance response to pembrolizumab (MK-3475) in microsatellite stable advanced colorectal cancer

**DOI:** 10.1186/s13148-023-01485-x

**Published:** 2023-04-29

**Authors:** Marina Baretti, Adrian G. Murphy, Marianna Zahurak, Nicole Gianino, Rose Parkinson, Rosalind Walker, Tamara Y. Lopez-Vidal, Lei Zheng, Gary Rosner, Nita Ahuja, Schalper Kurt, Nilofer S. Azad

**Affiliations:** 1 The Sidney Kimmel Comprehensive Cancer Center at Johns Hopkins, Baltimore, MD USA; 2 Yale School of Medicine, New Haven, CT USA

**Keywords:** Epigenetic drugs, Histone deacetylases inhibitors, DNA methyltransferases inhibitors, Colorectal cancer, Microsatellite proficiency, Immunotherapy

## Abstract

**Background:**

Approximately 95% of advanced colorectal cancer patients (CRC) have mismatch repair MMR-proficient (MMRp) tumors, which do not respond to PD1 blockade alone. Preclinical studies have shown that combined histone deacetylases (HDAC) and/or DNA methyltransferases (DNMT) inhibition can induce susceptibility to immune checkpoint therapy and inhibit tumor growth. We conducted a pilot trial evaluating PD-1 immune checkpoint inhibitor therapy in combination with DNMT and HDAC inhibitors in MMRp CRC. The study was designed with a biological endpoint of change in immune cell infiltration, to determine the optimal epigenetic combination that optimizes the tumor microenvironment. This trial was designed to test that hypothesis.

**Results:**

From January 2016 to November 2018, 27 patients were enrolled with median age of 57 (range 40–69) years. Median progression-free survival and overall survival were 2.79 months and 9.17, respectively. One patient in Arm C achieved a durable partial response by RECIST criteria, lasting for approximately 19 months. The most common treatment-related hematological adverse events in all arms were anemia (62%), lymphopenia (54%) and thrombocytopenia (35%), and non-hematological AEs were anorexia (65%), nausea (77%), and vomiting (73%).

**Conclusions:**

The combination of 5-azacitidine and romidepsin with pembrolizumab was safe and tolerable in patients with advanced MMRp CRC, but with a minimal activity. Further mechanistic investigations are needed to understand epigenetic-induced immunologic shift and to expand the potential applicability of checkpoint inhibitors in this setting.

**Supplementary Information:**

The online version contains supplementary material available at 10.1186/s13148-023-01485-x.

## Background

Colorectal cancer (CRC) is the second leading cause of cancer-related deaths, and over 2.2 million new cases and 1.1 million patient deaths are expected by 2030 [1]. Despite recent advances in methods and strategies to diagnose and treat CRC, there are still hurdles in the early diagnosis of this disease, and up to half of the patients present with metastatic CRC (mCRC). Standard chemotherapy combined with targeted therapy results in improved outcomes in patients with mCRC [2]. Nevertheless, the overall prognosis of mCRC is still suboptimal.

Cancer immunotherapy with immune checkpoint inhibitors, such as those targeting CTLA-4 and PD-1/PD-L1, has now revolutionized the field of oncology by prolonging survival of patients with rapidly fatal cancers [3]. However, the most compelling activity has been seen in 20–30% of solid tumor patients with immunogenic tumors such as mismatch repair-deficient (MMRd) cancers, cancers that naturally attract T cell infiltration (TIL) [4, 5]. CRC is a highly molecular heterogeneous disease, and only approximately 3–5% of metastatic CRC are considered microsatellite unstable (MSI-high)/MMRd [6, 7]. No objective clinical responses with anti-PD-1 or an anti-PD-L1 antibodies were observed in mismatch repair proficient/microsatellite stable (MMRp/MSS) CRC patients in the original studies of the agents [6, 7]. MMRp CRC tumors are characterized by low tumor-mutation burden, low neoantigen burden, and a paucity of T cells, thus considering immunologically “cold” [8, 9]. To unleash an optimal antitumor immune response in MMRp CRC, combinatorial therapeutics that combine immune checkpoints with other modalities are being developed.

Epigenetic changes, such as modifications of histones or altered patterns of DNA methylation within a gene promoter, are heritable and reversible changes in the pattern of gene expression mediated by mechanisms other than the alteration of the primary nucleotide sequence of a gene [10]. Recent work with epigenetic modulatory drugs has shown that these agents may be capable of altering the immunogenicity of the tumor microenvironment (TME) [10]. Abnormal DNA methylation by DNA methyltransferases (DNMTs) is rampant in cancer. Inhibition of DNMTs though agents such as 5-azacitidine results in restoration of gene function by reversing the suppression of transcription critical for the suppression of an immune response, as well as tumorigenesis [11]. In addition, many tumors have decreased levels of histone acetylation compared to normal tissue; histone deacetylation inhibitors (HDACis), such as romidepsin, have been demonstrated to restore gene expression of important genes involved in tumor suppression and immune regulation [12].

Combining DNMT and HDAC inhibition has a synergistic effect in inducing re-expression of tumor suppressors silenced by DNA methylation. Importantly, these biological effects include increasing tumor-associated antigen (TAA) presentation, changing polarity of T cell populations towards greater effector and less regulatory T-cells, increasing interferon production, and increasing B7H1 surface expression. These changes would be predicted to potentially convert non-immunogenic tumors to becoming sensitive to immunotherapy. Consistent with this hypothesis, HDACi and DNMTI have been widely shown to increase sensitivity to immune checkpoint inhibitors in multiple in vivo model systems [13, 14]. To test this hypothesis, we conducted a phase 1b, biomarker-driven trial, evaluating DNMT and HDAC inhibitors alone or in combination in sensitizing MMRp CRC to anti-PD1 therapy.

## Results

### Patients and treatment

**Fig. 1:**
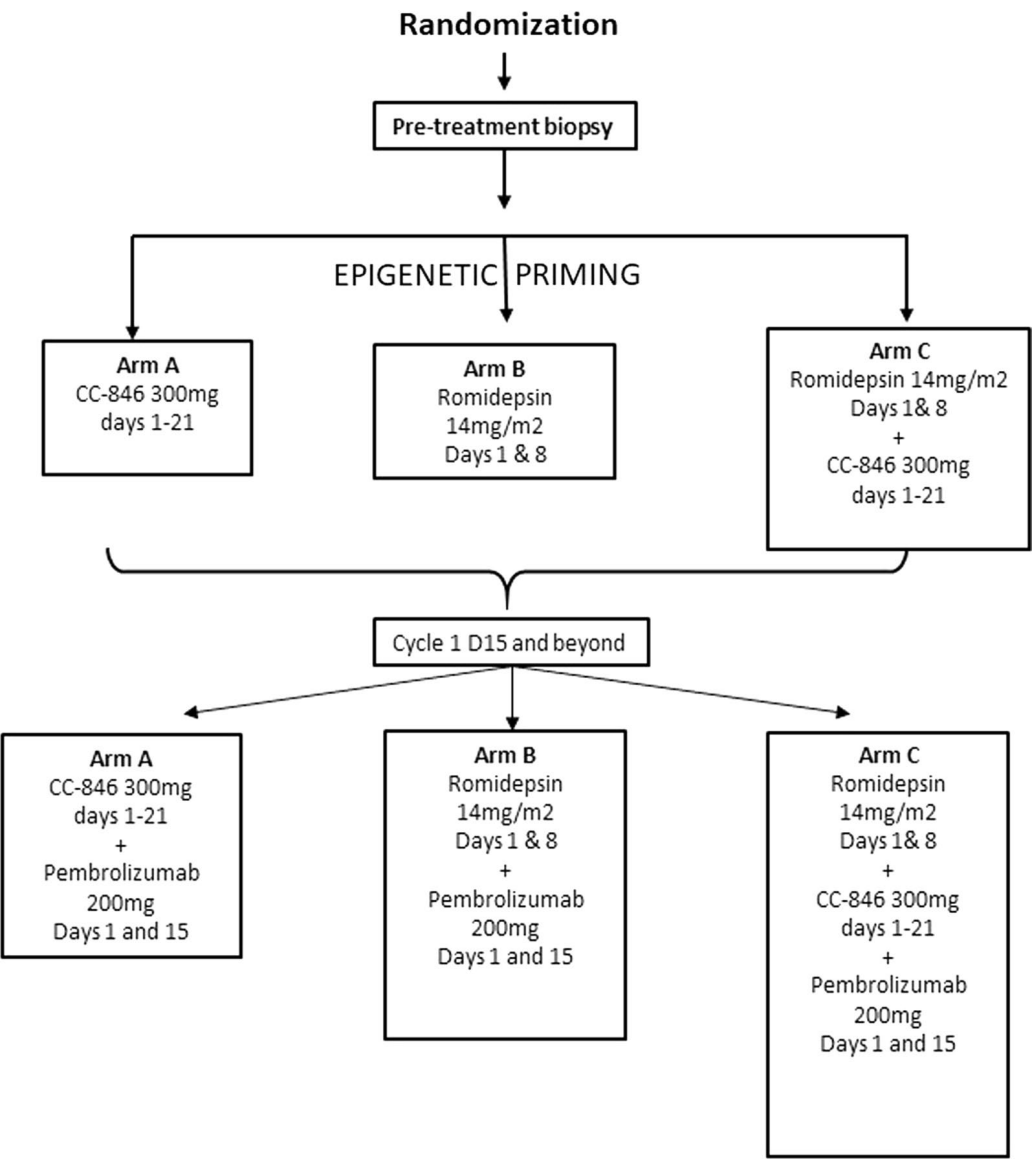
Trial design

Between February 2016 and November 2018, twenty-seven patients were randomized in the three treatment arms: Arm A (CC-486 + pembrolizumab *n* = 7), Arm B (Romidepsin + Pembrolizumab, *n* = 10), and Arm C (CC-486 + Romidepsin + Pembrolizumab, *n* = 10) (Fig. 1). Paired biopsies for 13 of 27 patients (48%) were obtained for the primary analysis: 4 of 7 (57%) patients in Arm A, 5 of 10 (50%) in Arm B and 4 of 10 (40%) in Arm C. All but one patient started on the planned therapy and were evaluable for toxicity endpoints. All patients were heavily pretreated, with a median of 2 prior therapies. The majority of patients discontinued treatment due to disease progression, either radiological (*n* = 17) or clinical (*n* = 6). Three patients discontinued the treatment due to toxicity (one due to acute kidney injury, one for posterior reversible encephalopathy syndrome, both possibly related to study drugs, and one for cardiac arrest unrelated to study drugs). Demographic and baseline parameters are given (overall and by study arm) in Table 1.

**Table 1:** Demographic and baseline characteristics

Variable	*N*	Missing	Aza	Rom	Aza + Rom	Overall	*p*
Histology, n (%)	27	0					1.00
Adenocarcinoma			7 (100.0)	10 (100.0)	10 (100.0)	27 (100.0)	
Age, median (min, max)	27	0	57.0 (48.0, 69.0)	49.0 (46.0, 67.0)	58.5 (40.0, 68.0)	57.0 (40.0, 69.0)	0.36
Gender, *n* (%)	27	0					0.39
Male			3 (42.9)	7 (70.0)	4 (40.0)	14 (51.9)	
Female			4 (57.1)	3 (30.0)	6 (60.0)	13 (48.1)	
Race, *n* (%)	26	1					0.59
White			6 (85.7)	5 (55.6)	6 (60.0)	17 (65.4)	
Black or AA			1 (14.3)	3 (33.3)	4 (40.0)	8 (30.8)	
Asian			0 (0.0)	1 (11.1)	0 (0.0)	1 (3.8)	
Ethnicity, *n* (%)	27	0					1.00
Non-Hispanic			7 (100.0)	9 (90.0)	10 (100.0)	26 (96.3)	
Hispanic or Latino			0 (0.0)	1 (10.0)	0 (0.0)	1 (3.7)	
Nodal status, *n* (%)	22	5					0.10
Negative			1 (16.7)	0 (0.0)	3 (42.9)	4 (18.2)	
Positive			5 (83.3)	9 (100.0)	4 (57.1)	18 (81.8)	
LN total, median (min, max)	27	0	21.0 (0.0, 28.0)	16.5 (0.0, 51.0)	14.5 (0.0, 62.0)	19.0 (0.0, 62.0)	0.83
Margin, *n* (%)	23	4					1.00
Negative			5 (83.3)	8 (80.0)	6 (85.7)	19 (82.6)	
Positive			1 (16.7)	2 (20.0)	1 (14.3)	4 (17.4)	
PNI, *n* (%)	16	11					0.66
Absent			2 (66.7)	2 (25.0)	2 (40.0)	6 (37.5)	
Present			1 (33.3)	6 (75.0)	3 (60.0)	10 (62.5)	
LVI, *n* (%)	17	10					1.00
Absent			1 (25.0)	2 (33.3)	3 (42.9)	6 (35.3)	
Present			3 (75.0)	4 (66.7)	4 (57.1)	11 (64.7)	
Site, *n* (%)	27	0					0.38
Right			1 (14.3)	3 (30.0)	5 (50.0)	9 (33.3)	
Transverse			0 (0.0)	0 (0.0)	0 (0.0)	0 (0.0)	
Left			5 (71.4)	4 (40.0)	2 (20.0)	11 (40.7)	
Rectum			1 (14.3)	3 (30.0)	3 (30.0)	7 (25.9)	
Grade, *n* (%)	24	3					0.74
Well			0 (0.0)	2 (20.0)	0 (0.0)	2 (8.3)	
Moderate			5 (83.3)	7 (70.0)	6 (75.0)	18 (75.0)	
Poor			1 (16.7)	1 (10.0)	2 (25.0)	4 (16.7)	
CEA, median (min, max)	27	0	10.2 (1.7, 1477.0)	97.2 (4.9, 415.4)	42.5 (4.1, 1033.3)	43.0 (1.7, 1477.0)	0.66
ANC, median (min, max)	27	0	3.75 (1.89, 19.69)	4.87 (2.90, 8.20)	3.20 (2.31, 5.81)	3.66 (1.89, 19.69)	0.25
ALC, median (min, max)	27	0	1.88 (0.72, 2.71)	1.37 (1.00, 2.50)	1.29 (0.49, 2.29)	1.31 (0.49, 2.71)	0.55
NLR, median (min, max)	27	0	2.46 (1.01, 8.56)	3.51 (1.57, 7.70)	2.51 (1.74, 6.96)	2.74 (1.01, 8.56)	0.81

### Limited efficacy was noted with therapy

**Fig. 2:**
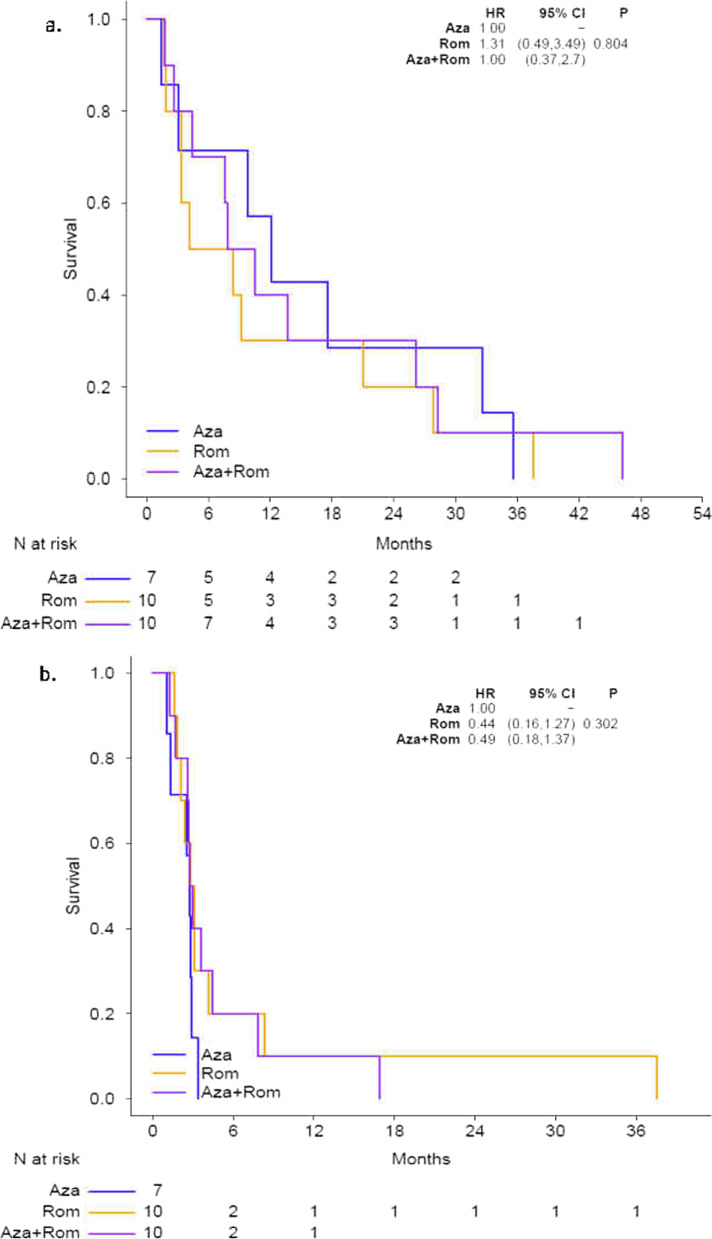
**a** Overall survival and **b** progression-free survival

The median time on treatment was 70 days (range 7–583 days). Nine patients discontinued treatment prior to disease reassessment by imaging and therefore were not assessable for response endpoints. Of the remaining 18 patients, one patient in Arm C achieved a durable partial response by RECIST criteria, lasting for approximately 19 months. One patient in Arm B achieved a best response of stable disease lasting 2.8 months, after which time the patient withdrew consent prior to any documented progression. Sixteen patients demonstrated progressive disease by the first scan (Table 2). Ten patients came off study before first restaging imaging was performed for clinical progression (*n* = 7) or toxicity (*n* = 1, acute kidney injury), while one patient withdrew consent; these patients were not considered evaluable per protocol. Progression-free survival (PFS) and overall survival (OS) curves by arm are shown in Fig. 2a, b**,** respectively.

**Table 2:** Best response per RECIST v1.1

Category	*N*	Denominator	Proportion (%)	Lcl (%)	Ucl (%)
PD	16	18	88.9	65.29	98.62
SD	1	18	5.6	0.14	27.29
PR	1	18	5.6	0.14	27.29
CR	0	18	0.0	0.00	18.53

### Combination of epigenetic modulation and immune checkpoint therapy is tolerable

Treatment-related adverse events are summarized in Table 3a and did not differ significantly by treatment arm. Overall, grade ≥ 3 drug-related adverse events (AEs) were encountered in 15 patients (58%) (Table 3b). The most common grade ≥ 3 drug-related toxicities pertained to hematologic toxicity in Arms A and B and included lymphopenia (29% and 22%, respectively) and anemia (14% and 44%). Importantly, neutropenia ≥ 3 was also observed in 29% of patients in Arm A, but none in Arm B.

**Table 3:** Overall drug-related adverse events (a) and grade 3 or higher drug-related adverse events (b)

System organ class Preferred term	Arm A (*N* = 7) Aza + Pembro (%)	Arm B (*N* = 9) Romi + Pembro (%)	Arm C (*N* = 10) Aza + Romi + Pembro (%)	Total (*N* = 26) (%)
(a)				
Blood and Lymphatic disorders	6 (86%)	8 (89%)	9 (90%)	23 (88%)
Anemia	3 (43%)	7 (78%)	6 (60%)	16 (62%)
Lymphocyte count decreased	2 (29%)	5 (56%)	7 (70%)	14 (54%)
Neutrophil count decreased	3 (43%)	1 (11%)	2 (20%)	6 (23%)
Platelet count decreased	0 (0%)	4 (44%)	5 (50%)	9 (35%)
White blood cell decreased	3 (43%)	0 (0%)	2 (20%)	5 (19%)
Endocrine disorders	0 (0%)	1 (11%)	2 (20%)	3 (12%)
Hyperthyroidism	0 (0%)	0 (0%)	2 (20%)	2 (8%)
Hypothyroidism	0 (0%)	1 (11%)	1 (10%)	2 (8%)
Gastrointestinal disorders	7 (100%)	8 (89%)	10 (100%)	25 (96%)
Abdominal pain	0 (0%)	0 (0%)	3 (30%)	3 (12%)
Anorexia	5 (71%)	5 (56%)	7 (70%)	17 (65%)
Ascites	1 (14%)	0 (0%)	1 (10%)	2 (8%)
Bloating	2 (29%)	1 (11%)	0 (0%)	3 (12%)
Constipation	1 (14%)	2 (22%)	0 (0%)	3 (12%)
Diarrhea	6 (86%)	1 (11%)	4 (40%)	11 (42%)
Dry heaves	0 (0%)	2 (22%)	1 (10%)	3 (12%)
Dry mouth	2 (29%)	0 (0%)	0 (0%)	2 (8%)
Dyspepsia	1 (14%)	2 (22%)	0 (0%)	3 (12%)
Dysphagia	1 (14%)	0 (0%)	0 (0%)	1 (4%)
Flatulence	1 (14%)	0 (0%)	0 (0%)	1 (4%)
Gastrointestinal reflux disease	0 (0%)	0 (0%)	1 (10%)	1 (4%)
Gastroparesis	1 (14%)	0 (0%)	0 (0%)	1 (4%)
Mouth pain	0 (0%)	0 (0%)	1 (10%)	1 (4%)
Mucositis oral	0 (0%)	1 (11%)	1 (10%)	2 (8%)
Nausea	6 (86%)	4 (44%)	10 (100%)	20 (77%)
Vomiting	4 (57%)	5 (56%)	10 (100%)	19 (73%)
Weight loss	2 (29%)	4 (44%)	3 (30%)	9 (35%)
General disorders	6 (86%)	9 (100%)	8 (80%)	23 (88%)
Chills	0 (0%)	3 (33%)	1 (10%)	4 (15%)
Edema limbs	1 (14%)	2 (22%)	2 (20%)	5 (19%)
Fatigue	5 (71%)	6 (67%)	8 (80%)	19 (73%)
Fever	2 (29%)	3 (33%)	4 (40%)	9 (35%)
Infections	2 (29%)	1 (11%)	2 (20%)	5 (19%)
Infection, tongue	0 (0%)	0 (0%)	1 (10%)	1 (4%)
Mucosal infection	0 (0%)	1 (11%)	0 (0%)	1 (4%)
Mucositis oral	2 (29%)	0 (0%)	1 (10%)	3 (12%)
Investigations	5 (71%)	6 (67%)	6 (60%)	17 (65%)
Alkaline phosphatase increased	3 (43%)	4 (44%)	5 (50%)	12 (46%)
ALT increased	3 (43%)	3 (33%)	2 (20%)	8 (31%)
AST increased	3 (43%)	1 (11%)	1 (10%)	5 (19%)
Blood bilirubin increased	2 (29%)	0 (0%)	0 (0%)	2 (8%)
Creatinine increased	1 (14%)	0 (0%)	3 (30%)	4 (15%)
INR increased	0 (0%)	1 (11%)	0 (0%)	1 (4%)
Prolonged QTC	1 (14%)	0 (0%)	0 (0%)	1 (4%)
Metabolism and nutrition disorders	4 (57%)	5 (56%)	8 (80%)	17 (65%)
Dehydration	0 (0%)	0 (0%)	1 (10%)	1 (4%)
Hyperglycemia	1 (14%)	4 (44%)	4 (40%)	9 (35%)
Hypermagnesemia	1 (14%)	1 (11%)	0 (0%)	2 (8%)
Hypernatremia	0 (0%)	0 (0%)	1 (10%)	1 (4%)
Hypoalbuminemia	0 (0%)	3 (33%)	3 (30%)	6 (23%)
Hypocalcemia	0 (0%)	2 (22%)	5 (50%)	7 (27%)
Hypokalemia	0 (0%)	1 (11%)	1 (10%)	2 (8%)
Hypomagnesemia	0 (0%)	0 (0%)	2 (20%)	2 (8%)
Hyponatremia	1 (14%)	2 (22%)	3 (30%)	6 (23%)
Hypophosphatemia	1 (14%)	4 (44%)	3 (30%)	8 (31%)
Musculoskeletal disorders	2 (29%)	2 (22%)	5 (50%)	9 (35%)
arthralgia	2 (29%)	0 (0%)	3 (30%)	5 (19%)
Arthritis	0 (0%)	0 (0%)	1 (10%)	1 (4%)
Back pain	2 (29%)	0 (0%)	0 (0%)	2 (8%)
Body aches	0 (0%)	0 (0%)	1 (10%)	1 (4%)
Myalgia	0 (0%)	0 (0%)	2 (20%)	2 (8%)
Pain in extremity	0 (0%)	2 (22%)	0 (0%)	2 (8%)
Nervous system disorders	1 (14%)	7 (78%)	6 (60%)	14 (54%)
Cognitive disturbance	0 (0%)	0 (0%)	1 (10%)	1 (4%)
Dizziness	0 (0%)	1 (11%)	1 (10%)	2 (8%)
Dysgeusia	1 (14%)	6 (67%)	4 (40%)	11 (42%)
Dysphasia	1 (14%)	0 (0%)	0 (0%)	1 (4%)
Headache	0 (0%)	2 (22%)	1 (10%)	3 (12%)
Paresthesia	0 (0%)	0 (0%)	1 (10%)	1 (4%)
Tremor	0 (0%)	0 (0%)	1 (10%)	1 (4%)
Psychiatric disorders	0 (0%)	1 (11%)	2 (20%)	3 (12%)
Anxiety	0 (0%)	0 (0%)	1 (10%)	1 (4%)
Insomnia	0 (0%)	0 (0%)	1 (10%)	1 (4%)
Irritability	0 (0%)	1 (11%)	0 (0%)	1 (4%)
Renal and urinary disorders	0 (0%)	1 (11%)	1 (10%)	2 (8%)
Chronic kidney disease	0 (0%)	0 (0%)	1 (10%)	1 (4%)
Proteinuria	0 (0%)	1 (11%)	0 (0%)	1 (4%)
Respiratory disorders	3 (43%)	2 (22%)	2 (20%)	7 (27%)
Cough	3 (43%)	1 (11%)	1 (10%)	5 (19%)
Dyspnea	0 (0%)	1 (11%)	1 (10%)	2 (8%)
Pleuritic pain	0 (0%)	1 (11%)	0 (0%)	1 (4%)
Rhinorrhea	0 (0%)	0 (0%)	1 (10%)	1 (4%)
Sore throat	0 (0%)	0 (0%)	1 (10%)	1 (4%)
Skin and subcutaneous disorders	3 (43%)	1 (11%)	1 (10%)	5 (19%)
Discoloration, hands	0 (0%)	0 (0%)	1 (10%)	1 (4%)
Erythema	0 (0%)	0 (0%)	1 (10%)	1 (4%)
Pruritus	1 (14%)	0 (0%)	1 (10%)	2 (8%)
Rash	3 (43%)	0 (0%)	1 (10%)	4 (15%)
Skin peeling	0 (0%)	1 (11%)	0 (0%)	1 (4%)
Vascular disorders	0 (0%)	2 (22%)	3 (30%)	5 (19%)
Diaphoresis	0 (0%)	0 (0%)	1 (10%)	1 (4%)
Hot flash	0 (0%)	1 (11%)	0 (0%)	1 (4%)
Hypertension	0 (0%)	0 (0%)	1 (10%)	1 (4%)
Hypotension	0 (0%)	0 (0%)	1 (10%)	1 (4%)
Night sweats	0 (0%)	1 (11%)	0 (0%)	1 (4%)
(b)				
Any grade3 + related AE	4 (57%)	7 (78%)	4 (40%)	15 (58%)
Blood and lymphatic disorders	4 (57%)	5 (56%)	2 (20%)	11 (42%)
Anemia	1 (14%)	4 (44%)	0 (0%)	5 (19%)
Lymphocyte count decreased	2 (29%)	2 (22%)	1 (10%)	5 (19%)
Neutrophil count decreased	2 (29%)	0 (0%)	1 (10%)	3 (12%)
Gastrointestinal disorders	0 (0%)	1 (11%)	2 (20%)	3 (12%)
Abdominal pain	0 (0%)	0 (0%)	1 (10%)	1 (4%)
Ascites	0 (0%)	0 (0%)	1 (10%)	1 (4%)
Diarrhea	0 (0%)	1 (11%)	0 (0%)	1 (4%)
Nausea	0 (0%)	0 (0%)	1 (10%)	1 (4%)
Vomiting	0 (0%)	0 (0%)	2 (20%)	2 (8%)
General disorders	0 (0%)	3 (33%)	0 (0%)	3 (12%)
Fatigue	0 (0%)	2 (22%)	0 (0%)	2 (8%)
Fever	0 (0%)	1 (11%)	0 (0%)	1 (4%)
Investigations	2 (29%)	1 (11%)	1 (10%)	4 (15%)
Alkaline phosphatase increased	1 (14%)	1 (11%)	1 (10%)	3 (12%)
ALT increased	1 (14%)	0 (0%)	0 (0%)	1 (4%)
AST increased	2 (29%)	0 (0%)	0 (0%)	2 (8%)
Blood bilirubin increased	1 (14%)	0 (0%)	0 (0%)	1 (4%)
Metabolism and nutrition disorders	0 (0%)	2 (22%)	1 (10%)	3 (12%)
Hyponatremia	0 (0%)	0 (0%)	1 (10%)	1 (4%)
Hypophosphatemia	0 (0%)	2 (22%)	0 (0%)	2 (8%)
Musculoskeletal disorders	0 (0%)	0 (0%)	1 (10%)	1 (4%)
Arthritis	0 (0%)	0 (0%)	1 (10%)	1 (4%)

The most common grade ≥ 3 drug-related toxicities in Arm C were gastrointestinal AEs (abdominal pain, nausea and vomiting observed in 10% of the patients), anemia and neutropenia, observed in 10% of patients. Treatment-related serious adverse events (Grade 3–4) occurred in 2 patients: one in Arm B (fever) and one in Arm C (vomiting), respectively. There were no treatment-related deaths.

### No significant change in CD8^+^ and CD8^+^:CD4^+^ ratio with treatment

A total of 22 patients underwent baseline tumor biopsies and, of those, 13 had matched pre- and post-treatment biopsies assessable for change in CD8^+^ TILs and the change in CD8^+^/CD4^+^ ratio. CD8^+^ T cell density is quantified by the number of nuclei staining positive for CD8 per mm^2^. Descriptive statistics for primary outcome data, overall and by treatment arm, are provided in Supplements (Additional file 1: Tables S1 and S2).

**Fig. 3:**
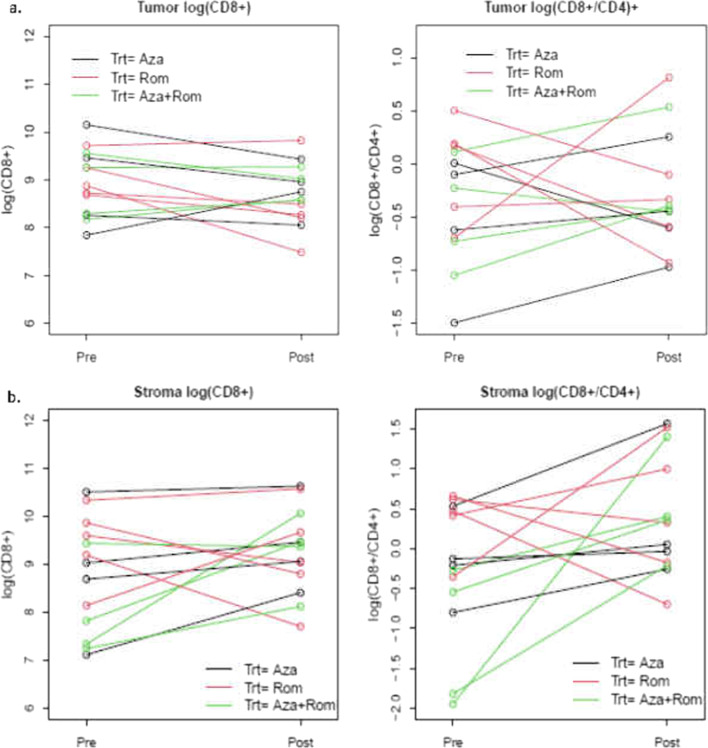
Plot of CD8^+^-positive cells per mm^2^ of tumor area and CD8^+^/CD4^+^ as quantified by immunohistochemistry in the tumor (**a**) and in the stroma (**b**). Matched baseline and post-treatment measurements are connected by a line. In total, 13 patients had matched post-treatment biopsies. Aza, azacitidine plus pembrolizumab (Arm A); Rom, romidepsin plus pembrolizumab (Arm B), Aza + Rom, azacitidine plus romidepsin plus pembrolizumab (Arm C)

Overall, we did not observe significant immunologic responses based on the pre-specified primary endpoint of an increase of the percentage of CD8^+^ TILs and/or the ratio of CD8^+^/CD4^+^ TILs in colorectal cancer tissue (Table 4). The mean changes in CD8^+^ and CD8^+^/CD4^+^ between arms in this study were also not significant, *p* = 0.29 and *p* = 0.64, respectively (Additional file 1: Table S3), though multiple patients (3/4 patients in Arm A and 2/5 patients in Arm C) did have an increase in the ratio in CD8^+^/CD4, suggestive of a possible biological effect in a subset of patients (Fig. 3).

**Table 4:** Paired *t* tests for the change in log(CD8^+^) and the log(CD8^+^/CD4^+^)

Group	Variable CI *t*	*n* complete both	Mean difference	*p* value *t* test
*Overall*				
Tumor log(CD8) [− 0.63, 0.12]	tumor.CD8	13	− 0.256	0.16
Tumor log(CD8/CD4) [− 0.37, 0.48]	tumor.CD8.CD4	13	0.054	0.79
*Aza*				
Tumor log(CD8) [− 1.28, 1.02]	tumor.CD8	4	− 0.13	0.74
Tumor log(CD8/CD4) [− 0.68, 0.91]	tumor.CD8.CD4	4	0.112	0.68
*Rom*				
Tumor log(CD8) [− 1.35, 0.16]	tumor.CD8	5	− 0.596	0.09
Tumor log(CD8/CD4) [− 1.47, 1.11]	tumor.CD8.CD4	5	− 0.181	0.72
*Aza + Rom*				
Tumor log(CD8) [− 0.62, 0.70]	tumor.CD8	4	0.043	0.85
Tumor log(CD8/CD4) [− 0.30, 0.88]	tumor.CD8.CD4	4	0.289	0.22

*Aza* azacitidine plus pembrolizumab (Arm A), *Rom* romidepsin plus pembrolizumab (arm B), *Aza + Rom* azacitidine plus romidepsin plus pembrolizumab (Arm C)

**Fig. 4:**
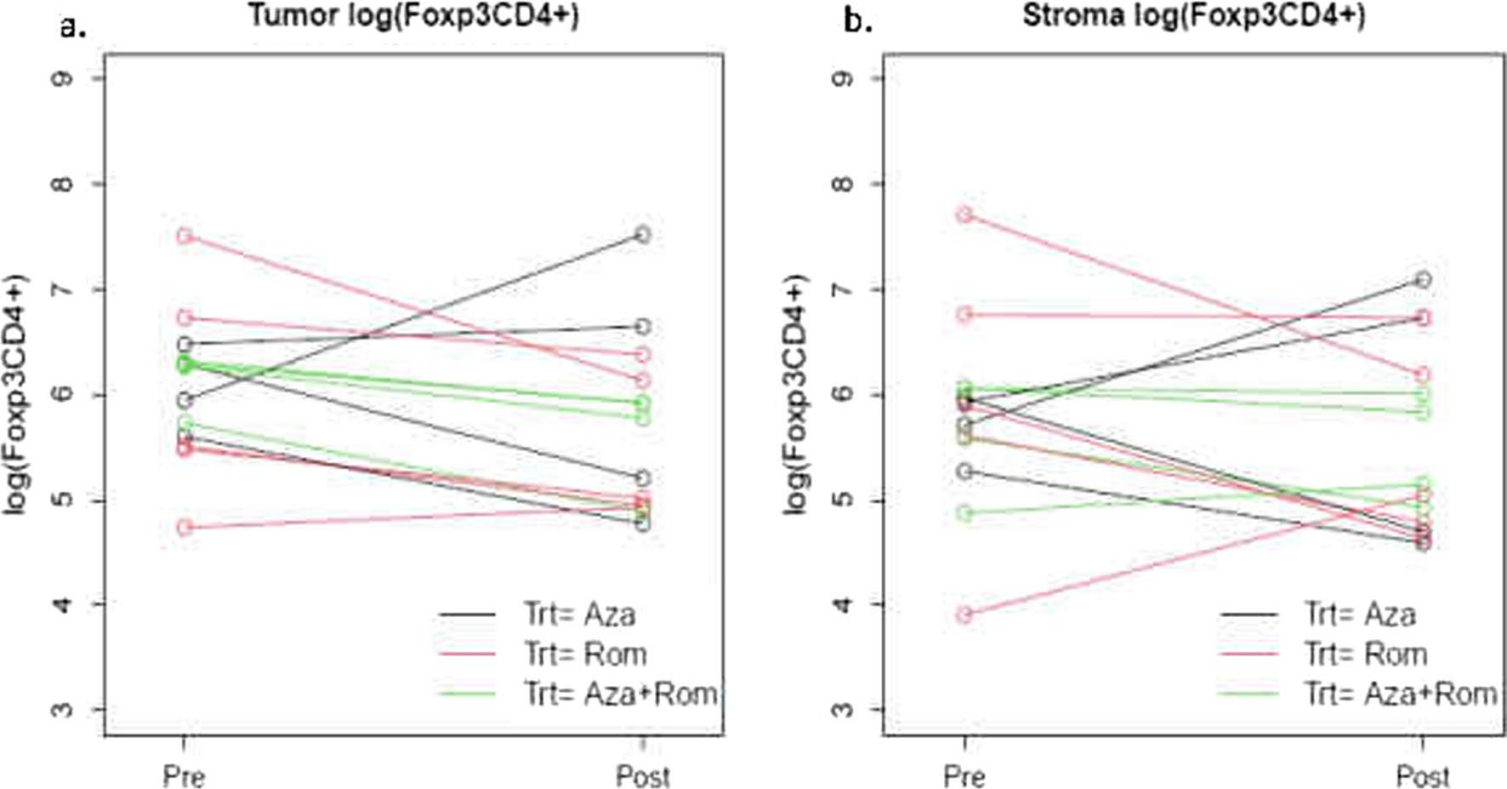
Plot of Foxp3-positive CD4 T cells per mm2 of tumor area as quantified by immunohistochemistry in the tumor (**a**) and in the stroma (**b**). Matched baseline and post-treatment measurements are connected by a line. In total, 13 patients had matched post-treatment biopsies. Aza, azacitidine plus pembrolizumab (Arm A); Rom, romidepsin plus pembrolizumab (Arm B), Aza + Rom, azacitidine plus romidepsin plus pembrolizumab (Arm C)

Interestingly, we observed a trend toward a decrease in tumor regulatory Foxp3CD4^+^ cells in response to treatment in the overall populations (*p* = 0.09). This decrease was more pronounced in patients who received romidepsin and achieved statistical significance in the combination arm of romidepsin plus azacitidine plus immunotherapy (*p* = 0.02) (Table 5 and Fig. 4).

**Table 5:** Summary of CD8^+^, CD4^+^ and FOXp3^+^ T cell in pre- and post-treatment biopsies, as recorded in tumor, and in the stroma

Group	Variable CI *t*	*n* complete both	Mean difference	*p* value *t* test
Overall				
Tumor log(CD8) [− 0.63, 0.12]	tumor.CD8	13	− 0.256	0.16
Tumor log(CD8/CD4) [− 0.37, 0.48]	tumor.CD8.CD4	13	0.054	0.79
Tumor Foxp3 [− 0.81, 0.07]	tumor.Foxp3	13	− 0.371	0.09
Stroma Foxp3 [− 0.79, 0.34]	stroma.Foxp3	13	− 0.225	0.40
*Aza*				
Tumor log(CD8) [− 1.28, 1.02]	tumor.CD8	4	− 0.13	0.74
Tumor log(CD8/CD4) [− 0.68, 0.91]	tumor.CD8.CD4	4	0.112	0.68
Tumor Foxp3 [− 1.96, 1.88]	tumor.Foxp3	4	− 0.04	0.95
Stroma Foxp3 [− 1.91, 2.03]	stroma.Foxp3	4	0.058	0.93
*Rom*				
Tumor log(CD8) [− 1.35, 0.16]	tumor.CD8	5	− 0.596	0.09
Tumor log(CD8/CD4) [− 1.47, 1.11]	tumor.CD8.CD4	5	− 0.181	0.72
Tumor Foxp3 [− 1.21, 0.19]	tumor.Foxp3	5	− 0.513	0.11
Stroma Foxp3 [− 1.84, 0.84]	stroma.Foxp3	5	− 0.501	0.36
*Aza + Rom*				
Tumor log(CD8) [− 0.62, 0.70]	tumor.CD8	4	0.043	0.85
Tumor log(CD8/CD4) [− 0.30, 0.88]	tumor.CD8.CD4	4	0.289	0.22
Tumor Foxp3 [− 0.88, − 0.18]	tumor.Foxp3	4	− 0.525	0.02
Stroma Foxp3 [− 0.78, 0.45]	stroma.Foxp3	4	− 0.164	0.46

## Discussion

Emerging data support that epigenetically targeted agents have major immunomodulatory effects and may be ideal agents to convert non-intrinsically immunotherapy-sensitive tumors into being susceptible to immunomodulatory agents [15]. Here, we report the results of a trial testing DNMT and HDAC inhibitors as sensitizers of MMR-p CRC to anti-PD1 therapy. Unfortunately, a clinical effect of the regimen was not appreciated, with most patients experiencing progressive disease by the first scan. Nonetheless, one patient had a very durable, RECIST confirmed, partial response and another achieved a durable stable disease as best response. In a population where the expected response rate to checkpoint inhibition is virtually zero, the above responses suggest a possible modulatory effect with epigenetic priming in a small subset of patients. As such, important questions remain regarding whether concurrent targeting of other pathways can further enhance the clinical benefit of this combination in CRC.

Preclinical data in colon and breast murine models from Zhou et al. showed remarkable synergy when epigenetic modulators were combined with agents that block immune checkpoints [13]. Of note, in their model, they used both anti-CTLA-4 and anti-PD-1 inhibitors and found that the addition of either the class I specific HDAC inhibitor, entinostat, or DNMT inhibitor, azacitidine (5-AZA), significantly decreased metastases and tumor growth, and increased survival. Mechanistic studies showed that tumor-infiltrating CD8^+^ T cells (assessed by flow cytometry) increased by approximately fourfold after PD-1/CTLA-4 inhibition. Inclusion of 5-AZA and entinostat in the treatment regimen did result in a significant decrease in tumor-infiltrating FoxP3^+^ Regulatory T cells (Tregs) and circulating granulocytic myeloid-derived suppressor cells (G-MDSCs), compared to either untreated tumors or tumors treated with immune checkpoint blockade alone. These data expand and support the work from other groups highlighting the Tregs-targeting and immune-promoting effect of HDAC inhibition [16, 17]. For instance, in a murine renal cell carcinoma model and in castration-resistant prostate cancer, Pili et al. [18] showed that entinostat, in combination with either IL-2, survivin-based vaccine therapy, or immune checkpoint inhibition, reduced Foxp3 levels in Tregs, and this was associated with enhanced tumor growth inhibition. In gastrointestinal cancers, data from our group in a pancreatic preclinical model showed that the addition of dual checkpoint inhibition to HDAC inhibitor led to improved infiltration of activated CD8^+^ T effector cells and increased cytotoxic characteristics, dependent on decreased functionality of immunosuppressive MDSCs [19]. These studies [18–20] suggest that the known effect of immune checkpoint blockade, which lead to expansion and increased activity of cytotoxic effector T cells, may not be sufficient in many tumors unless immune-suppressor cells are reduced, potentially by treatment with epigenetic modulators. This study showed modest benefit for a small subset of patients on the trial, but arguably its greatest value was validating, in humans, the previous murine preclinical data showing the biological effect of epigenetic agents on the immunosuppressive Foxp3 T regulatory cell compartment. Of note, the decrease in Tregs, as defined by positive FOXp3 expression, was statistically significant in HDAC inhibitor containing arms, but not in Arm A (azacitidine plus pembrolizumab). These data suggest that the effect on Tregs was driven by HDACi exposure, as has been previously demonstrated in the literature, with the azacitidine adding no benefit [13]. Moreover, emerging clinical data are showing the utility of HDAC inhibition to reverse primary and secondary immune resistance in cutaneous and uveal melanoma as well as non-small cell lung cancer, further increasing excitement about this strategy [21, 22].

This study has several limitations, including limited tissue availability after the samples were used to assess the primary endpoint. As such, further analysis concerning pharmacokinetics (PK) and pharmacodynamic (PD) analysis and inferences concerning the epi-transcriptome profile were not conducted. We do acknowledge this is of utmost important and would be great explorations in future studies. However, prior studies have assessed the effects of the drugs used in the study, looking a same dose and schedule. For instance, PK and/or PD data were available for 59 patients receiving CC-486 dosing schedules at 300 mg once-daily (or 200 mg twice-daily for 14 or 21 days) per 28-day cycle. Both 300 mg once-daily schedules and the 200 mg twice-daily 21-day schedule significantly (all *p* < 0.05) reduced global DNA methylation in whole blood at all measured time points (days 15, 22, and 28 of the treatment cycle), with sustained hypomethylation at cycle end compared with baseline. CC-486 exposures and reduced DNA methylation were significantly correlated [23–25]. Similarly, PK and PD data for romidepsin at the dose chosen for the trial are available showing higher or maintained histone acetylation post-therapy [26–28]. Furthermore, gene-expression profiling using patient-derived samples has been incorporated in some studies and showed that genes involved in apoptosis, cell proliferation, immune regulation and angiogenesis are altered as early as 4 h after drug administration [26, 29–31]. We have accordingly selected these approved doses of CC-486 and romidepsin for this study using the above data. From a clinical trial design perspective, important novel aspects of trial design should be highlighted. This study was designed as a phase 0 study with a co-primary biological endpoint, CD8^+^ TILS or the ratio of CD8/CD4. The trial design was intended to allow investigators to assess multiple combinations for their biological effect, prior to taking a combination further in larger sample size trials. This kind of novel design will grow more and more important as the drug development community grapples with the need to test seemingly limitless combination strategies with limited patients. Indeed, while the trial failed to show significant clinical activity, the biological endpoints suggest that future immunotherapy combinations with HDAC inhibitors are reasonable as a strategy to modulate regulatory T cells. As our immune-oncology armamentarium expands, better understanding of the biological impact on the TME of the tools we have, will allow us to design scientifically informed combination strategies.

This trial highlights the complexity of combining epigenetic agents with immunotherapy. First, the non-specificity of epigenetic agents and their effect on multiple transcriptional events create major challenges in identifying biomarker predictors of benefit. In melanoma and other solid tumors, intratumoral CD8^+^ T cell infiltration, both before and during treatment, has been associated with response to checkpoint blockade. This study had 2 patients with prolonged disease benefit. A key step for this approach would be to identify a biomarker to better determine which patients will respond to a combination HDACi + PD1i strategy. Secondly, multiple dosing strategies could be used that could impact the immune response; concurrent therapy versus sequencing epigenetic therapy before or after the immune checkpoint inhibitor should be addressed. As it remains unknown whether appropriate sequencing of epigenetic agents and immune checkpoint inhibitors therapy may be important for the induction of optimal antitumor immunity, future preclinical work should continue to address this question, as well as novel trial designs that allow for testing various sequences of therapies [10, 32, 33].

## Conclusion

In summary, this study provides evidence that the combination of DNMT and HDAC inhibitors to pembrolizumab is safe and tolerable in patients with CRC. The clinical activity observed does not suggest that these regimens by themselves should be advanced further. However, the evidence of substantial clinical benefit for a small subset of patients and significant decreases in Tregs supports possible investigation on targeting other pathways to enhance the benefit of this combination in CRC.

## Methods

### Study design and endpoints

This was an open-label, single-institution, phase 1b trial conducted at the Sidney Kimmel Comprehensive Cancer Center (SKCCC) at John Hopkins University (JHU) to evaluate the safety and feasibility of a DNMT inhibitor, HDAC inhibitor, or both, in combination with pembrolizumab and its immunological effects using an immune endpoint of change in changes CD8^+^ and/or the ratio of CD8^+^/CD4^+^ Tils on pre- and post-treatment biopsies.

The study included three treatment arms and was designed with a safety run in for each. The first three patients randomized to Arms A and C received CC-486 (oral azacitidine) 300 mg daily for a total of 21 (Arm A) or 14 (Arm C) days, respectively. Dosing after the first three patients was halted to allow for safety evaluation before treating additional patients. If 2 or more of the first 3 patients experienced a dose-limiting toxicity (DLT), CC-486 dose would have been reduced to 300 mg daily for 14 days in Arm A and to 200 mg daily for 14 days in Arm C (Fig. 1).

The primary clinical objective of the study was to evaluate the safety and tolerability of DNMT inhibitor (CC-486), an HDAC inhibitor (romidepsin), or both, in combination with pembrolizumab in patients with advanced MSS CRC. Primary translational endpoint was to characterize changes in CD8- and CD4-positive tumor-infiltrating lymphocytes (TILs) by in pre- and post-treatment tumor specimens.

Secondary objectives included progression-free survival (PFS), overall survival (OS). Explorative objectives included overall response rate (ORR) and change in gene expression and methylation in immune gene signaling circuits.

The study protocol was approved by the institutional review board of the Johns Hopkins School of Medicine and was conducted according to the Declaration of Helsinki and the guidelines for Good Clinical Practice. All patients signed a written informed consent before the conduct of any study procedures and after a full explanation of the study to the patient by the study investigator. The trial was registered under ClinicalTrials.gov as (NCT02512172).

### Study procedures

Treatment was administered on 28-day cycles and consisted of: oral CC-486 300 mg days 1–21 + pembrolizumab 200 mg i.v. days 1 and 15, every 28 days (Arm A); romidepsin 14 mg/m^2^ i.v. days 1, 8 and 15 + pembrolizumab 200 mg i.v days 1 and 15, every 28 days (Arm B); oral CC-486 300 mg days 1–14 plus romidepsin 8 mg/m^2^ i.v. days 8 and 15 plus pembrolizumab 200 mg i.v days 1 and 15, every 28 days (Arm C).

The dose of CC-486 was based on previous studies showing that extending CC-486 dosing to 21 day did not alter PK as compared to SC azacitidine 75 mg/m^2^ administered for the first 7 days of a 28-day cycle. Due to the short plasma half-life of azacitidine, daily CC-486 dosing over 14 or 21 days showed no evidence of drug accumulation, nor was there evidence of decreased absorption after multiple doses. Moreover, extending CC-486 dosing to 14 or 21 days sustains methylation reductions over the entire treatment cycle [23]. The approved dose of romidepsin is 14 mg/m^2^ days 1, 8, and 15 of 28-day cycles. In patients with T cell lymphomas who received romidepsin at this dose and schedule, the geometric mean values of the maximum plasma concentration (Cmax) and the area under the plasma concentration versus time curve (AUC0-inf) were 377 ng/mL and 1549 ng * h/mL, respectively [26–28]. Further studies have evaluated population pharmacokinetic model of romidepsin in patients with cutaneous or relapsed peripheral T-cell lymphoma who received a 4-h infusion at the dose of 14 or 18 mg/m^2^ during their first treatment cycle. The disposition of romidepsin was well characterized by the two-compartment model with a linear elimination and exhibited moderate inter-patient variabilities [24].

We have accordingly selected these doses of CC-486 and romidepsin for this study.

The study included a 14-day lead-in treatment with an epigenetic agent (CC-486 and/or romidepsin). After the lead in with epigenetic therapy, patients concurrently received the epigenetic agent plus pembrolizumab 200 mg every 2 weeks until the time of progression, unacceptable toxicity or withdrawal of consent. All patients underwent fresh tumor biopsy at baseline, and on combination therapy after cycle 2 day 15 but before cycle 3 day 1.

### Patients' selection

Patients were eligible for the trial if they were 18 years or older with histologically proven adenocarcinoma of the colon or rectum (mCRC) that was metastatic who had progressed to at least one prior line of treatment for metastatic CRC including fluoropyrimidines, oxaliplatin and/or irinotecan.

Mismatch repair proficiency/microsatellite stability (MSS) phenotype had to be assessed by a CLIA-certified laboratory using a CLIA-certified assay for microsatellite testing or immunohistochemistry for MMR proteins. Eastern Cooperative Oncology Group (ECOG) performance status ≤ 1, and adequate organ function as defined by absolute neutrophil count ≥ 1500 cells/μL, platelet count ≥ 100,000 cells/μL, AST and ALT ≤ 3 × upper limit of normal (or ≤ 5 × upper limit of normal in patients with liver metastases), total bilirubin ≤ 1.5 × upper limit of normal, and serum creatinine within normal institutional limits or creatinine clearance ≥ 60 mL/min. Patients with primary refractory disease (progression at the first restaging during first line therapy), those with known history or evidence of brain metastases, prior treatment with checkpoint inhibitors, autoimmune disease, uncontrolled intercurrent illness, or other contraindications to receive the study drugs or their components were excluded. Disease amenable to biopsy with acceptable clinical risk was required for participation. Measurable disease according to RECIST 1.1 criteria was also required.

### Assessments

Patients were evaluated every cycle for trial therapy compliance and monitoring of adverse events. The National Cancer Institute Common Terminology Criteria for Adverse Events (CTCAE) version 4.0 was implemented for adverse event monitoring. The treatment protocol allowed dose delays or reduction if patients experienced unacceptable side effects and adverse reactions related to study drug(s). Events classified as possibly, probably, or definitely related by the study investigator were considered treatment-related. Radiographic assessments of response were performed every 3 cycles (approximately 12 weeks and analyzed for response according to Response Evaluation Criteria in Solid Tumors (RECIST) version 1.1. In the event that the patient was deemed to be receiving continued clinical benefit in the face of progressive disease by RECIST criteria, the patient may have continued on therapy with agreement of the Principal Investigator. If progressive disease was confirmed on successive imaging or clinical examination, the date of progression was marked as the first timepoint that progression was noted. Upon progression of disease, patients were monitored for long-term adverse events and survival.

### Quantitative multiplex immunofluorescence (QIF)

Previously validated and standardized quantitative multiplex immunofluorescence protocol using specific antibodies and serial control tissue microarray (TMA) sections was used to measure antigen expression. In brief, freshly cut histology sections from each case were deparaffinized and subjected to antigen retrieval using EDTA buffer (pH = 8.0) and boiled for 20 min at 97 °C (PT Module, Lab Vision). Slides were then incubated with dual endogenous peroxidase block (#S2003, DAKO) for 10 min at room temperature and subsequently incubated with a blocking solution containing 0.3% bovine serum albumin in 0.05% Tween-20 for 30 min. Slides were simultaneously stained with pan-cytokeratin (clone AE1/AE3, eBioscience), FoxP3 (D2W8E, Cell Signaling Technology), CD8 (clone C8/144B, DAKO), CD4 (clone SP35, Abcam) and 4′, 6-Diamidino-2-Phenylindole (DAPI) for visualization of all cell nuclei. Secondary isotype-specific HRP-conjugated antibodies and tyramide-based fluorescent reagents were subsequently added for the signal detection of each marker, including anti-rabbit Envision (K4003, DAKO) with Cy5-tyramide (Akoya Biosciences), anti-mouse IgG1 antibody (Abcam) with Cy3-tyramide (Akoya Biosciences), and goat anti-rabbit IgG antibody (Abcam) with biotinylated tyramide/Streptavidin-AlexaFluor750 conjugate (Invitrogen). Residual horseradish peroxidase activity between sequential detection incubations with secondary antibodies was eliminated by exposing the slides twice with a solution containing 100 mM benzoic hydrazide and 50 mM hydrogen peroxide in phosphate-buffered solution.

### Fluorescence signal quantification

Quantitative measurement of fluorescence signal was performed using the AQUA® method, which generates objective and sensitive measurements of each target within the user-defined tissue compartments. In brief, the quantitative immunofluorescence (QI)F score of each marker expression was defined by the signal detected in the tumor compartment (cytokeratin-positive cells), stromal compartment (cytokeratin-negative cells), and the total tissue area signal detected in the whole sample (DAPI-positive total tissue compartment). Scores were normalized to the exposure time and bit depth for each captured image, allowing all collected scores to be comparable.

### Statistical methods

The primary statistical endpoints of this study were change in CD8^+^ TILs and change in CD8^+^/CD4^+^ ratio. CD8^+^ T cell density is quantified by the number of nuclei staining positive for CD8 per mm^2^. The target accrual goal was 24 evaluable patients, 8 per treatment arm. To account for patients being not evaluable, up to 30 patients could have been enrolled.

Data are summarized on the natural scale with descriptive statistics (mean, standard deviation, median, and range) overall and for each arm separately. Following log transformation, changes in log(CD8^+^) and log(CD8^+^/CD4^+^) TILs were evaluated with paired t-tests, and comparisons of changes between arms made with ANOVA.

Analysis of safety and efficacy endpoints were performed on all patients who received at least one dose of study drug. Adverse events and toxicity were classified and graded according to the Common Toxicity Criteria for Adverse Events (CTCAE) version 4.0. Kaplan–Meier curves were used to estimate probabilities of progression free survival (PFS) and overall survival (OS). Cox proportional-hazards models were used to compare PFS and OS between treatment arms. All statistical tests were two-sided, and *p* values < 0.05 were considered significant. All statistical analyses were performed using R version 3.5.3.

## Supplementary Information

**Additional file 1:** **Table S1.** CD8^+^ by study arm (natural scale). Aza, azacitidine plus pembrolizumab (Arm A); Rom, romidepsin plus pembrolizumab (arm B); Aza + Rom, azacitidine plus romidepsin plus embrolizumab (Arm C). **Table S2.** CD8^+^/CD4^+^ by study arm (natural scale). Aza, azacitidine plus pembrolizumab (Arm A); Rom, romidepsin plus pembrolizumab (arm B); Aza + Rom, azacitidine plus romidepsin plus pembrolizumab (Arm C). **Table S3**. ANOVA log(CD8^+^) and log(CD8^+^/CD4^+^) changes. Aza, azacitidine plus pembrolizumab (Arm A); Rom, romidepsin plus pembrolizumab (arm B); Aza + Rom, azacitidine plus romidepsin plus pembrolizumab (Arm C).

## Data Availability

The datasets used and/or analyzed during the current study are available from the corresponding author upon reasonable request.

## Abbreviations

5 = AZA: Azacitidine
AEs: Adverse events
CRC: Colorectal cancer
CTCAE: Common Toxicity Criteria for Adverse Events
DNMT: DNA methyltransferases
G-MDSCs: Granulocytic myeloid-derived suppressor Cells
HDAC: Histone deacetylases
MDSCs: Myeloid-derived suppressor cells
MMRp: Mismatch repair proficient
MMRd: Mismatch repair deficient
MSI: Microsatellite instability
MSS: Microsatellite stable
OS: Overall survival
PFS: Progression-free survival
TAA: Tumor-associated antigen
TIL: T cell infiltration
TME: Tumor microenvironment
Tregs: Regulatory T cells
