# Supplementary material for: A study of using epigenetic modulators to enhance response to pembrolizumab (MK-3475) in microsatellite stable advanced colorectal cancer
Source: Clin Epigenetics. 2023 Apr 29;15:74. doi: 10.1186/s13148-023-01485-x (PMC10149019; doi:10.1186/s13148-023-01485-x)

**Supplements**

**Table S1. CD8+ by study arm (natural scale)**

*Aza, azacitidine plus pembrolizumab (Arm A); Rom= romidepsin plus pembrolizumab (arm B), Aza+Rom, azacitidine plus romidepsin plus pembrolizumab (Arm C)***.**


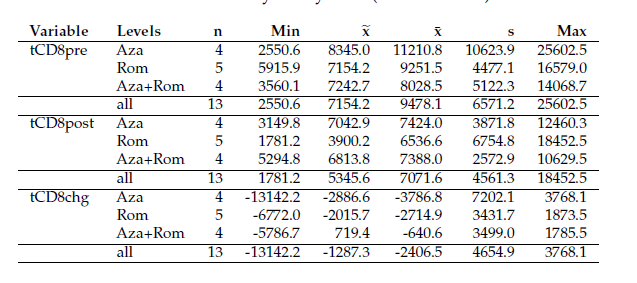


**Table S2. CD8+/CD4+ by study arm (natural scale)**

*Aza, azacitidine plus pembrolizumab (Arm A); Rom= romidepsin plus pembrolizumab (arm B), Aza+Rom, azacitidine plus romidepsin plus pembrolizumab (Arm C)***.**


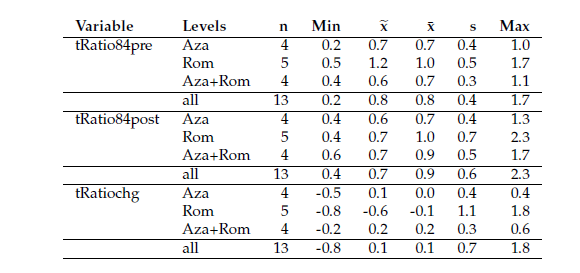


**Table S3. ANOVA log(CD8+) and log(CD8+/CD4+) changes**

*Aza, azacitidine plus pembrolizumab (Arm A); Rom= romidepsin plus pembrolizumab (arm B), Aza+Rom, azacitidine plus romidepsin plus pembrolizumab (Arm C)***.**


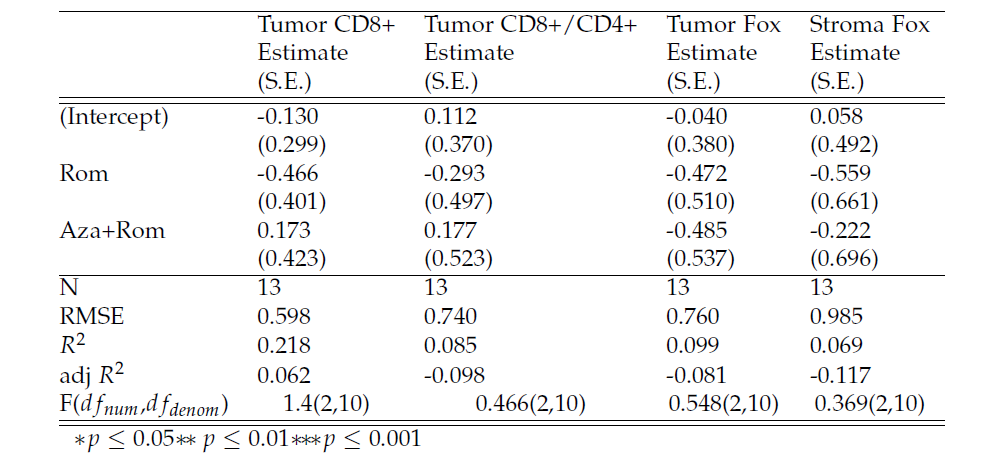

Supplement: Supplementary file 1 — Additional file 1: Table S1. CD8+ by study arm (natural scale). Aza, azacitidine plus pembrolizumab (Arm A); Rom, romidepsin plus pembrolizumab (arm B); Aza + Rom, azacitidine plus romidepsin plus embrolizumab (Arm C). Table S2. CD8+/CD4+ by study arm (natural scale). Aza, azacitidine plus pembrolizumab (Arm A); Rom, romidepsin plus pembrolizumab (arm B); Aza + Rom, azacitidine plus romidepsin plus pembrolizumab (Arm C). Table S3. ANOVA log(CD8+) and log(CD8+/CD4+) changes. Aza, azacitidine plus pembrolizumab (Arm A); Rom, romidepsin plus pembrolizumab (arm B); Aza + Rom, azacitidine plus romidepsin plus pembrolizumab (Arm C). [file 13148_2023_1485_MOESM1_ESM.docx]
